# The Need for Well-Educated Probationers

**Published:** 1909-05-08

**Authors:** 


### Nursing Administration.

### THE NEED FOR WELL-EDUCATED PROBATIONERS.

In the announcements displayed in nursing papers for probationers the stipulation is now almost invariably made that applicants must be "well educated." The larger general hospitals do not of course need to advertise. It is the cottage hospitals, the fever and smaller special institutions which sometimes make known their wants in this way, and the striking change which has taken place in nursing during the last ten years is emphasised by the announcement that even for these posts, which cannot in the nature of things lead on to the highest prizes in the nursing world, "good education is essential." The phrase is to some extent intended as protective. The matron who advertises is overwhelmed with utterly unsuitable applications, and hopes to guard herself from the rush of inadequate candidates by letting it be known that a certain degree of aptitude for learning is necessary. The term is relative, and implies in effect very little. But it does express the common preference accorded by matrons to women who have profited by mental training and are qualified as regards memory and caligraphy to understand and transmit written instructions, and acquire without too much difficulty an unfamiliar nomenclature. There can be no doubt that the disposition to engage only such probationers as are capable of taking an intelligent interest in their work marks a great advance in nursing ideals. It need not be regarded as an attempt to discredit those whose education has been conducted in the elementary schools. The girl who has passed the seventh standard is probably far better prepared to listen to lectures and take notes than the girl who has had a private governess or spent years at an expensive boarding school. In the elementary schools the instruction given approximates far more closely to the kind of teaching available for the hospital probationer than does that at the private school. The classes are large, the amount of individual attention which it is possible to bestow is small, and the pupils are compelled to do a great deal for themselves. The system which suffices to prepare girls and boys to enter secondary schools and take scholarships for training colleges, and even for the universities, ought to turn out good probationers, but the great evil of elementary education is that it stops short at the most critical moment in the development. However satisfactorily examinations may have been passed at the age of fourteen or fifteen, the woman who has not received any consecutive instruction since is not in a very good position at the age of twenty-two or twenty-three for acquiring the elements of a science such as physiology, with which she is altogether unfamiliar. The extent to which knowledge fades when not constantly exercised is well known to all who have ever taught. Allow seven years during which the only mental training has been listening to sermons, or reading penny novelettes, and the habit of consecutive thought, and of concentrated attention, with the power of accurate expression on paper, will probably have become much impaired. These are the causes which induce matrons to invite the application of "well educated" probationers, meaning, not probationers who have attended any particular kind of school, but probationers who have continued their studies to an age when it is more likely that a permanent impression has been made on them.

It is becoming one of the most pressing problems of the nursing world how to secure the best class of women for hospital training. The hospitals are under grave disadvantages in their search for good material. Owing to the age at which it is found advisable to receive probationers, they are dependent first on the leisured classes, who can never furnish sufficient material; and, secondly, on the wastage from other professions. It is not a satisfactory position of affairs. It is a question whether some effort should not be made to induce the educational authorities to cater for the requirements of the hospitals in their evening continuation schools. Girls often make up their minds to become nurses long before there is any possibility of commencing training in any well-equipped hospital. Could not the opportunity be given them in special courses of study to prepare themselves beforehand for the studies they will be required to master when they begin their systematic training? The County Councils have shown themselves ready to meet the case so far as they can appreciate the need. They are only too anxious to spread a knowledge of home nursing among the senior scholars, and to provide lecturers on nursing subjects for mothers of families. But they have never had the need brought before them of preparing the ground for the instruction to be given in the training schools. Lectures on nursing are worse than useless for girls who want to become probationers. But classes on the construction of the human frame, on the elements of hygiene, and on elementary cookery would prove the best possible introduction to the probationer's course, and we feel sure that the County Councils would follow the lead of any hospital authorities who would take the trouble to indicate what they want from candidates. It is a little hard on the candidate who wishes to procure training to be told that the passport to it is a "good education" and to leave the standard wholly nebulous. Suppose a woman wants to become a nurse who is well aware that she has not had what any reasonable person would be justified in calling a good education, might she not be given the chance of making up for deficiencies? When all is undefined the most worthy may be discouraged out of sheer humility, and the self-satisfied candidate may succeed by pertinacity. We should like to see a preliminary course of evening instruction open to women who want to qualify for probationerships in every large town, by means of which those whose education stopped short too early could rub up their scholarship, and the first tremendous difficulties in the way of mastering unfamiliar studies could be overcome.

But in order to make such instruction really useful co-operation between the hospitals and the County Councils is essential.

